# Prevalence and correlates of health anxiety among medical students: a cross-sectional study from the United Arab Emirates

**DOI:** 10.1186/s43045-022-00273-2

**Published:** 2023-01-11

**Authors:** Karim Abdel Aziz, Emmanuel Stip, Afra Al-Sanadi, Alreem Al-Shamsi, Hessah Al-Sharqi, Mariam Eisa Al-Zaabi, Noora Al-Shehhi, Dina Aly El-Gabry

**Affiliations:** 1 Department of Psychiatry, College of Medicine and Health Sciences, United Arab Emirates University, Al-Ain, United Arab Emirates; 2 Institut Universitaire en Santé Mentale de Montréal & CHUM, (Université de Montréal Health Centre), University of Montreal, Montreal, Canada; 3 Department of Psychiatry, Sheikh Khalifa Medical City, Abu Dhabi, United Arab Emirates; 4 Ophthalmology Department, Sheikh Khalifa Medical City, Abu Dhabi, United Arab Emirates; 5 Neuropsychiatry Department, Faculty of Medicine, Okasha Institute of Psychiatry, Ain Shams University, Cairo, Egypt

**Keywords:** Health anxiety, Illness anxiety, Hypochondriasis, Medical students, Medical students’ syndrome

## Abstract

**Background:**

It is often reported that medical students repeatedly develop health anxiety related to the diseases that they are studying. To the best of our knowledge, health anxiety has not been investigated in medical students in the United Arab Emirates (UAE). Therefore, we aimed to investigate the prevalence of health anxiety among a sample of medical students attending the United Arab Emirates University (UAEU). We conducted a cross-sectional study of 193 undergraduate medical students (68 males, 125 females) across the 6 years of the College of Medicine at the UAEU. Students were screened for health anxiety using the Short Health Anxiety Inventory (SHAI).

**Results:**

Eighteen students (9.3%) reached the threshold for clinically significant health anxiety on the SHAI (score ≥ 27). There was no statistically significant difference between those with and those without health anxiety in age, gender, place of origin, or year of study. There was a statistically significant difference (*p* < 0.05) between the two groups as regards a past history of medical or mental health conditions influencing their choice of college. No specific student demographic or background characteristics significantly predicted the occurrence of clinically significant health anxiety.

**Conclusions:**

Health anxiety was prevalent in a significant proportion of subjects in our sample (almost one in every ten students). Individual experiences of medical and mental illness may play a role in the development of health anxiety and in the choice of studying medicine.

## Background

Health anxiety (HA) refers to a preoccupation with or fear of having a serious illness, which is often triggered by a misinterpretation of bodily sensations and is associated with repeated medical checking that usually only provides transient reassurance [1–4]. It is suggested that medical students repeatedly develop health anxiety related to the diseases that they are studying, a phenomenon sometimes referred to as “medical student syndrome” [5].

Health anxiety involves two main components that include cognitions (the ideas and thoughts that one may have a particular illness) and the anxiety triggered by the cognitions [6]. It has been demonstrated that medical students experience large amounts of psychological distress due to the quantity of work involved in studying, the stress of examinations, the competitive environment, and the anxiety associated with new clinical experiences [7]. Stress is thought to enhance physical sensations through activating the autonomic nervous system, making individuals more aware of their bodies [8]. This stress, combined with the intense exposure to medical knowledge, is thought to affect expectations about illness beliefs, leading to selective attention to specific bodily sensations [9]. Medical knowledge can also demonstrate the fine line between health and illness, leading to a reconceptualization of previously neglected symptoms within the context of newly obtained knowledge [8].

Studies of the prevalence of health anxiety in medical compared to non-medical students have been mixed with some showing higher rates in medical students [6], others showing lower rates [10], while other studies show comparable rates [11, 12]. While transient symptoms of health anxiety are common in medical students, the evidence that it translates into increased numbers of consultations, and that clinicians should take it into account when assessing patients who are medical students, is weak [6]. While this might be explained by health anxiety symptoms not reaching a threshold for clinical significance, it might also reflect the reluctance of medical students to seek help for mental health issues. In a study of medical students carried out in the United Arab Emirates (UAE), about 69% reported that they thought most people would think less of someone who had received mental health treatment, reflecting a perceived negative public stigma towards mental health issues [13]. Therefore, health anxiety might be under-reported and under-recognized in the medical student population in the UAE. This is significant considering that the distress associated with health anxiety may interfere with the academic performance of medical students [12]. Given that there have been no previous studies from the UAE, screening for health anxiety in medical students would be relevant in order to identify whether symptoms reached a clinical threshold. Therefore, we aimed to investigate the prevalence and pattern of health anxiety in a sample of medical students attending the United Arab Emirates University (UAEU) and to investigate factors predicting clinically significant health anxiety. We hypothesized that demographics, educational year, and past and family illnesses would be different in those with compared to those without health anxiety.

## Methods

### Participants

We conducted a cross-sectional study that aimed to investigate the prevalence and pattern of health anxiety in a sample of medical students attending the College of Medicine and Health Sciences (CMHS), United Arab Emirates University (UAEU). Our study included 193 subjects who were recruited between September 2016 and April 2017. We included male and female subjects, aged 18 years or older, and who were medical students from across the six undergraduate years of the CMHS. The CMHS is located in the city of Al Ain in the eastern region of the emirate of Abu Dhabi (the largest of the seven emirates in the UAE) and is the oldest national medical school in the UAE [14]. The CMHS accepts students from all of the seven emirates and at the time of the study had an undergraduate student population of around 609 students [15]. Adequate sample size was calculated to be 151 subjects, based on a student population size of 609, a population proportion of 11% (based on results from previous studies about average rates of health anxiety in medical students), a 5% margin of error, and a 95% confidence interval.

### Tools

Data was collected for each subject’s demographic characteristics (age, gender, year of study, place of origin), previous higher education, years repeated at university, years taken out from college, medical or mental health conditions before starting university, and whether medical or mental health conditions in the student or someone close to them influenced their choice of college. Each subject was asked to complete the Short Health Anxiety Inventory (SHAI) [3], which is a reliable and valid self-rated measure of health anxiety that is sensitive to a spectrum of intensity of symptoms that range from mild concern to actual hypochondriasis. It also differentiates between people who suffer from health anxiety and those who have actual physical illness but who are not excessively concerned about their health. The SHAI consists of 18 questions with each question rated from 0 to 3, with a maximum score of 54. It consists of two sections: a main section evaluating the symptoms of health anxiety and a negative consequences section that looks at the individual’s perception of how dreadful it would be to be sick. These two sections are combined to give a total score, with a cutoff of ≥ 27 indicating clinically significant health anxiety [16].

### Procedures

All those meeting the inclusion criteria and consenting to participate were included. Written informed consent was obtained which included discussion of the aim of the research, that participation was totally free and voluntary, that participation did not have any impact on treatment, that participants were kept anonymous, that results would be used for scientific purposes only, and that any candidate could exit the study at any time without giving justification. Each subject was then asked to complete the information sheet and the Short Health Anxiety Inventory to assess for symptoms of health anxiety.

### Statistical analyses

Data was recorded and analyzed using the Statistical Package of Social Sciences IBM SPSS Statistics for Windows, version 26.0 (2019) (IBM Corp., Armonk, NY, USA). Normality of distribution of data for quantitative variables was confirmed using a quantile–quantile (Q-Q) plot curve that showed their distribution against the expected normal distribution. The results were tabulated, grouped, and statistically analyzed using the following tests: mean and standard deviation (SD) for parametric numerical (quantitative) data; frequency and percentage for nonnumerical (qualitative) data; chi-square test for comparing categorical variables; independent-sample student *t*-test to assess the statistical significance between two study group means; and logistic regression to measure the effect of factors that affect the occurrence of clinically significant health anxiety among medical students. A *p*-value of < 0.05 was considered statistically significant.

## Results

Of the 193 subjects who participated, 125 (64.8%) were females and 68 (35.2%) were males. The mean age of the sample was 20.61 years (*SD* ± 2.050). Of these, 40 (20.7%) were first-year students, 28 (14.5%) were second-year students, 23 (11.9%) were third-year students, 46 (23.8%) were fourth-year students, 28 (14.5%) were fifth-year students, and 28 (14.5%) were sixth-year students. Thirty-three students (17.1%) originated from Al-Ain. Using the SHAI, the mean total score for the whole sample was 16.63 (*SD* ± 6.777), the mean main section score was 13.89 (*SD* ± 5.957), and the mean negative consequences score was 2.74 (*SD* ± 2.105). Nineteen students (9.3%) reached the threshold for clinically significant health anxiety (SHAI total score ≥ 27). There was no statistically significant difference in demographic characteristics between students with health anxiety and students without health anxiety (Table 1).

**Table 1:** Demographic characteristics of students with and without health anxiety

	**With health anxiety** **(*N* = 19)**	**Without health anxiety** **(*N* = 174)**	**Test**	***p*-value**
**Mean age (SD)**	20.60 (± 2.069)	20.71 (± 1.929)	*t* = − 0.209	0.564
**Gender**				
*Male*	9 (47.4%)	59 (33.9%)	*χ*^2^ = 1.360	0.244
*Female*	10 (52.6%)	115 (66.1%)		
**Origin from Al-Ain**	2 (10.5%)	31 (17.8%)	*χ*^2^ = 2.008	0.366
**Not from Al-Ain**	17 (89.5%)	143 (82.2%)		
**Year of study**				
*First year*	1 (5.3%)	39 (22.4%)	*χ*^2^ = 5.643	0.343
*Second year*	4 (21.1%)	24 (13.8%)		
*Third year*	3 (15.8%)	20 (11.5%)		
*Fourth year*	4 (21.1%)	42 (24.1%)		
*Fifth year*	2 (10.5%)	26 (14.9%)		
*Sixth year*	5 (26.3%)	23 (13.2%)		

*N*, Number, *SD* Standard deviation, *t* Student *t*-test, *χ*^2^ chi-square test

Students with health anxiety had statistically significantly higher HAI total, main section, and negative consequences scores than students without health anxiety (*p* < 0.05) (Table 2). There was a statistically significant difference (*p* < 0.05) between the two groups as regards past history of medical or mental health conditions influencing the student’s choice of college (Table 2).

**Table 2:** Clinical characteristics and background of students with and without health anxiety

	**With health anxiety** **(*N* = 19)**	**Without health anxiety** **(*N* = 174)**	**Test**	***p*-value**
**SHAI score**	30.05 (± 2.778)	15.17 (± 5.312)	*t* = − 12.02	< 0.0001*
**Main section score**	12.67 (± 4.805)	25.05 (± 3.274)	*t* = − 10.94	< 0.0001*
**Negative consequences score**	2.49 (± 1.908)	5.00 (± 2.517)	*t* = − 5.256	< 0.0001*
**Previous higher education**	0 (0%)	6 (3.5%)	*χ*^2^ = 0.676	0.411
**Retaken any years at university**	0 (0%)	26 (14.9%)	*χ*^2^ = 3.281	0.070
**Taken years out from university**	0 (0%)	4 (2.3%)	*χ*^2^ = 0.446	0.504
**Past history of medical conditions**	5 (26.3%)	31 (17.8%)	*χ*^2^ = 0.816	0.366
**Past history of mental health problem**	2 (10.5%)	4 (2.3%)	*χ*^2^ = 3.849	0.050
**Past history of medical or mental health problem contributed to the choice of college**	3 (15.8%)	7 (4.0%)	*χ*^2^ = 4.827	0.028*
**Anyone close suffered from a serious health problem**	11 (57.9%)	82 (47.1%)	*χ*^2^ = 0.796	0.372

*N* Number, *SD* Standard deviation, *SHAI* Short Health Anxiety Inventory, *t* Student *t*-test, *χ*^2^ chi-square test

^*^statistically significant

Two students with health anxiety (both males) had a history of mental health problems (both had anxiety and depression), and 4 students without health anxiety (2 males, 2 females) had a history of mental health problems (one male had dysthymia and the other obsessive–compulsive disorder, one female had trichotillomania and the other had anxiety). Of the 3 students with health anxiety and whose past history of a medical or mental health problem contributed to their choice of college choice of college (all males), one had a history anxiety and depression, one had a history anxiety and depression and a history of prediabetes, and one had a history of avascular necrosis requiring a hip replacement.

Logistic regression analysis was carried out on demographic and students background characteristics to evaluate whether these factors predicted the occurrence of clinically significant health anxiety among medical students in our sample. The regression coefficient shows the effect of each variable after controlling the effect of other variables in the model. None of the variables entered into the model was significant predictors for the occurrence of clinically significant health anxiety (SHAI total score ≥ 27) (Table 3).

**Table 3:** Factors predicting the occurrence of health anxiety

	***B***	***S.E***	***OR***	**95% *CI***		***p*-value**
				**Lower**	**Upper**	
**Age**	− 0.054	0.382	0.947	0.448	2.001	0.887
**Gender**	0.381	0.548	1.464	0.500	4.286	0.487
**Year of study**	0.231	0.409	1.260	0.565	2.808	0.572
**Previous higher education**	− 19.044	15,598.592	< 0.0001	< 0.0001	-	0.999
**Retaken any years at university**	− 19.256	8238.959	< 0.0001	< 0.0001	-	0.998
**Taken years out from university**	− 17.864	19,097.234	< 0.0001	< 0.0001	-	0.999
**Past history of medical conditions**	− 0.086	0.654	0.918	0.255	3.305	0.895
**Past history of mental health problems**	− 18.909	18,597.869	< 0.0001	< 0.0001	-	0.999
**Past history of medical or mental health problem contributed to the choice of college**	0.770	1.231	2.159	0.193	24.120	0.532
**Anyone close suffered from a serious health problem**	0.429	0.543	1.535	0.530	4.449	0.430

*B* Regression coefficient, *SE* Standard error of the coefficient, *OR* Odds ratio, 95% *CI*, 95% confidence interval for the odds ratio

## Discussion

Our study found that 9.3% of participating medical students had clinically significant health anxiety (using the SHAI). We also found that a significantly higher rate of subjects with health anxiety had a history of medical or mental health problems contributing to their choice of college compared to those without health anxiety, although none of the students’ demographic and background characteristics significantly predicted the occurrence of clinically significant health anxiety. Rates of health anxiety in medical students have generally been high, although these have varied across studies. Early studies from the 1960s indicated prevalence rates of 70% and 78.8% in medical students [17, 18]. A later study by Kellner et al. [19] showed that these early rates were probably an exaggeration when they demonstrated a rate of 8.3%, which is comparable to our finding of 9.3%. Since then, findings from other countries have also been comparable to our study: 11.0% in Iran [20], 11.9% in Pakistan [21], and 16.1–17.4% in Saudi Arabia [22, 23]. The study in Norway by Ellingsen and Wilhelmsen [24] showed a rate of 1.25% that was lower than our finding. The variation in rates probably demonstrates methodological differences in measuring health anxiety across medical students in terms of the tools used and the sample sizes of different studies. Yet most of these rates in medical students seem to be comparable to rates of health anxiety in the general population, which has been reported to be between 2.1 and 13.1% [25].

However, the verdict on whether health anxiety is more common in medical students compared to non-medical students is less conclusive [6, 10–12]. Howes and Salkovskis [12] reported that rates of health anxiety seem to be no higher in medical students compared to other (non-medical) students and non-students. Similarly, Kellner et al. [19] reported no difference in rates of health anxiety in medical students compared to law students, as did Waterman and Weinman [26] who reported no difference in rates of health anxiety or the number of doctors’ visits made for new health issues since starting university between medical students, nonmedical science students, and law students. However, Moss-Morris and Petrie [6] found that both first- and third-year medical students scored higher than third-year law students on cognitive aspects of health anxiety, but that these differences did not translate into differences in the number of health visits in the previous year. Contrary to this, Ellingsen and Wilhelmsen [24] found that medical students had significantly lower health anxiety than law students and suggested that people without medical knowledge are more likely to interpret symptoms as something potentially serious and become anxious, while people with medical knowledge can probably better appraise the situation over time. They found no difference in the frequency of doctors’ visits.

Our study found that there was no significant difference between those with and those without health anxiety in terms of age, gender, year of study, whether they are originally from Al-Ain (where the university is based), previous higher education, any years retaken at university, or any years taken out of university. This seems to suggest that these are not significant factors in our sample as to whether medical students develop health anxiety or not. This is similar to Zahid et al. [21] who found that age, gender, year of medical school, and visits to the doctor in the previous 6 months were not associated with an increased risk of developing significant health anxiety. Ellingsen and Wilhelmsen [24] also found no gender difference in rates of health anxiety in medical students, as did Al-Turki et al. [27], who in addition to finding no gender differences also found no differences between early-year students compared to clinical students in rates of health anxiety. However, Moss-Morris and Petrie [6] found that first-year medical students scored higher on emotional distress and hypochondriacal concerns than third-year medical students, although they were comparable on the cognitive aspects of health anxiety. They suggested that health anxiety in medical students can be separated into a perceptual (cognitive) component and an emotional distress component, and that differentiating between these components may help explain the variations between different levels of medical students [6]. This may suggest that more senior medical students are better at appraising and managing distress associated with the idea they may have a health issue. Yet, Azuri et al. [28] noticed that among first- to sixth-year medical students, there was a significant rise in health anxiety and emotional distress on entering clinical years which decreased later on, but that the perceptual-cognitive aspects increased gradually over the 6 years without decline. Interference with life scores remained low all through the 6 years, suggesting that health anxiety depended on the year of study, but that it most probably did not interfere with students’ ability to function. Similarly, Eslami et al. [20] reported that rates of health anxiety were significantly higher in interns (15.3%) compared to clerkship students (8.1%), supporting the notion of rising health anxiety with progressing years. Contrary to this, Althagafi et al. [22] reported that preclinical students had significantly higher rates of health anxiety (21.1%) than clinical year students (14%), but rates in medical students did not significantly differ from non-medical students.

The fact that there was no significant difference in the number of students repeating years or taking years out from university seems to suggest that even in those who develop health anxiety, the impact on academic performance was not to a degree that delayed the progress of students from one year to another. However, this does not rule out the possibility that the academic performance of students with health anxiety was not affected compared to those without health anxiety. Although medical students with health anxiety in our sample may appear to be progressing similar to their peers, they may have still experienced a decline in their performance, but not to the degree that they repeated the year or take time out of university, suggesting that if academic performance is affected, then it is probably only to a mild or moderate degree.

Our study found no difference between those with and without health anxiety in terms of frequency of anyone close having suffered from a serious health problem, again suggesting that this was not a significant factor in our sample. On the contrary, Ellingsen and Wilhelmsen [24] reported that students who had experienced illness in close family tended to have lower health anxiety.

A significantly higher percentage of students in our sample with health anxiety had a history of medical or mental health problems contributing to the choice of college than those without health anxiety. Also in our sample, a near significantly (*p* = 0.05) higher percentage of students with health anxiety had a past history of mental health problems than those without health anxiety. Ellingsen and Wilhelmsen [24] found that students who reported a history of depression had significantly higher (*p* < 0.01) health anxiety scores compared to those without health anxiety. This suggests that perhaps the negative and pessimistic thinking that is commonly associated with depression could predispose individuals to negatively interpret physical symptoms and make them more prone to health anxiety. The near significant difference (*p* = 0.05) in those with and without health anxiety in terms of past history of mental health problems could possibly explain the significant difference between the two groups that a past history of medical or mental health conditions in the student influenced their choice of college. Individual experiences of medical and mental illness may play a role in the development of health anxiety and in the choice of studying medicine.

Although our study is the first to investigate health anxiety in the UAE, it was conducted before the onset of the covid-19 pandemic. So, our study provided a baseline regarding levels of health anxiety before the era of covid-19 where worldwide there have been increased concerns and worries in the public perception towards health issues. Since then, there have been a number of studies from the UAE looking at anxiety in university students, although none specifically measured health anxiety during this period. Saddik et al. [29] investigated a sample of 719 medical students across 4 emirates within the UAE in the first month of the lockdown in the UAE (March 2020) and found that 24.3% reported mild to severe anxiety on the GAD-7. Medical students reported lower anxiety levels compared to dental students and higher levels of anxiety during their clinical rotations which decreased with the introduction of online learning [29]. This represented a slight increase from the previous finding in medical students in the UAEU from before the covid-19 pandemic, which showed rates of anxiety (also using the GAD-7) of 20.7% [13], and from the previous finding by Awadalla et al. [30] that reported rates of anxiety of 22.3% (also on the GAD-7) in 404 undergraduate students at Zayed University, Dubai, before the covid-19 pandemic, although their sample did not include any medical students.

The study by Drissi et al. [31] also in the first month of the lockdown in the UAE found rates of anxiety and depression of 42.9% using the GHQ-12 in a sample of 154 students in the UAEU, although only 10 participants (6.5%) were medical students. Saravanan et al. [32] in the third month of the lockdown in the UAE (May 2020) reported rates of anxiety specifically related to covid-19 of 15.9% in a sample of 433 university students in Sharjah using the Coronavirus Anxiety Scale, of whom only 10.2% (*N* = 44) were medical students.

## Conclusions

Our study demonstrated that the rate of health anxiety in medical students in the UAEU was 9.3%, which was comparable to other studies, and that a significantly higher percentage of those with health anxiety had a history of physical and mental health problems contributing to their choice of college than those without health anxiety. While a significant number of medical students had health anxiety, it did not seem to delay their academic progress. In addition, compared to more recent rates in the UAE of other anxiety disorders during the covid-19 pandemic, there appears to be a trend towards increased anxiety in the context of concerns and public perceptions regarding global health issues.

## Data Availability

The datasets used and/or analyzed during the current study are available from the corresponding author on reasonable request.

## Abbreviations

CMHS: College of Medicine and Health Sciences
GAD-7: Generalized Anxiety Disorder Assessment
GHQ-12: General Health Questionnaire
HA: Health anxiety
SHAI: Short Health Anxiety Inventory
SD: Standard deviation
SPSS: Statistical Package for the Social Sciences
UAE: United Arab Emirates
UAEU: United Arab Emirates University
